# Interchangeability of patellar height measurement using the Insall–Salvati ratio at 0° and 30° of knee flexion during weightbearing in total knee arthroplasty

**DOI:** 10.1186/s43019-025-00294-1

**Published:** 2025-10-08

**Authors:** Sang Jun Song, Hyun Woo Lee, Cheol Hee Park

**Affiliations:** Department of Orthopaedic Surgery, Kyung Hee University College of Medicine, Kyung Hee University Medical Center, 23 Kyunghee-Daero, Dongdaemun-Gu, Seoul, 02447 Korea

**Keywords:** Knee, Patellar height, Insall–Salvati ratio, Flexion

## Abstract

**Purpose:**

To analyze the correlation and degree of agreement between the patellar heights measured at knee flexion of 0° and 30° before and after total knee arthroplasties (TKA) without patellar resurfacing.

**Methods:**

One hundred primary TKAs with nonresurfaced patella were prospectively evaluated. We measured the Insall–Salvati ratio (ISR), modified ISR (mISR), Blackburne-Peel ratio (BPR), and Caton-Deschamps ratio (CDR) in weightbearing true lateral radiograph at knee flexion of 0° and 30°. The correlations between the patellar height measured at knee flexion of 0° and 30° were analyzed by Pearson correlation analysis. The degree of agreement between the patellar heights in knee flexion angles of 0° and 30° was analyzed using the limits of agreement (LoA) of Bland–Altman analysis; a difference of <  ± 0.2 between the measurements at knee flexion of 0° and 30° was deemed clinically acceptable.

**Results:**

Very strong correlations existed between the pre- and postoperative ISR (r = 0.826 and 0.823, *p* < 0.001, respectively), and the preoperative mISR (*r* = 0.802, *p* < 0.001) measured between knee flexion angles of 0° and 30°. Strong correlation was observed in the other pre- and postoperative measurements. The range between upper and lower LoAs of the ISR measured at knee flexion of 0° and 30° was <  ± 0.20 preoperatively (−0.174 ~ 0.155) and postoperatively (−0.181 ~ 0.174), while the ranges for all other measurements did not lie within the clinically acceptable range.

**Conclusions:**

The ISR measured at 0° or 30° of knee flexion during weightbearing can be interchanged reasonably before and after TKA. Although knee flexion of 30° is the standard, lateral radiograph of 0° flexion can be a reasonable alternative for evaluating patellar height using the ISR.

*Level of Evidence:* Level II.

## Introduction

Patellar height is the general term used to describe the patellar position in the sagittal plane. Numerous patellar height parameters have been described, including the Insall–Salvati ratio (ISR) [1], the modified ISR (mISR) [2], the Blackburne-Peel ratio (BPR) [3], and the Caton-Deschamps ratio (CDR) [4]. Most involve a ratio between a measure of patellar length and a measure of the distance between an aspect of the patella and a landmark on the tibia [5]. The use of a ratio rather than an absolute length compensates for variations in radiographic magnification [5].

As a rule, the patellar height should be measured on a lateral radiograph at a knee flexion angle of 30° to ensure that the patellar tendon is stretched appropriately [1, 3]. However, to evaluate the severity of knee osteoarthritis or the postoperative condition following total knee arthroplasty (TKA), the lateral radiographs are typically obtained with the fully extended knee during weightbearing; it can be obtained relatively easily even in patients with loss of knee motion or severe pain restricting adequate flexion in arthritic and post-TKA conditions. The radiograph at 30° of knee flexion for patellar height evaluation is obtained only when patellofemoral-specific concerns, such as anterior knee pain or extensor lag, are present. Considering the radiation exposure and cost effectiveness, it is unclear whether it is necessary to check the lateral radiographs of 0° and 30° knee flexion together. Additionally, even though physicians take lateral radiographs routinely at a knee flexion angle of 30°, the majority of them could be taken at flexion angles ranging from 20° to 45° [6]. It appears that taking weight bearing true lateral radiographs in the fully extended position of the knee would be advantageous for maintaining consistency. Therefore, we wondered if the radiographic parameters for the patellar height measured in the weightbearing lateral radiographs taken at knee flexion angles of 0° and 30° were interchangeable.

The present study analyzed the correlation and degree of agreement between the patellar heights measured at knee flexion angles of 0° and 30° before and after TKA. We hypothesized that certain patellar height parameters would be interchangeable in the lateral radiographs taken at knee flexion angles of 0° and 30°.

## Materials and methods

### Materials

A prospectively nonrandomized consecutive study was designed. Patients, who underwent primary TKAs for late-stage osteoarthritis between May 2018 and March 2019, were enrolled. The inclusion criteria were primary TKA with patellar nonresurfacing owing to Kellgren–Lawrence grade 4 degenerative osteoarthritis with varus deformities. The exclusion criteria were inflammatory arthritis; a history of knee infection, fracture, dislocation, or ligament injury; history of high-tibial osteotomy; and/or flexion contracture. According to the criteria, 100 knees (78 patients) were included. The patients’ demographics are shown in Table 1. The study was approved by our Institutional Review Board. Informed consent was obtained from all patients before this study and no patient refused to participate in this study.

**Table 1:** Patient demographics

Number of knees (patients)	100 (74)
Age (years)	71.6 ± 6.2
Female/male	57/17
Body mass index (kg/m^2^)	26.5 ± 4.7
Right/left	46/54
Attune CR/Attune PS/Persona CR/Persona PS^*^	11/33/28/28
Follow-up period (years)	2.3 ± 0.2

^*^Types of prostheses implanted, including retaining (CR) and substituting (PS) the posterior cruciate ligament; Attune (Depuy, Raynham, MA) and Persona (Zimmer, Warsaw, Indiana)

### Evaluation of the patellar height

**Fig. 1:**
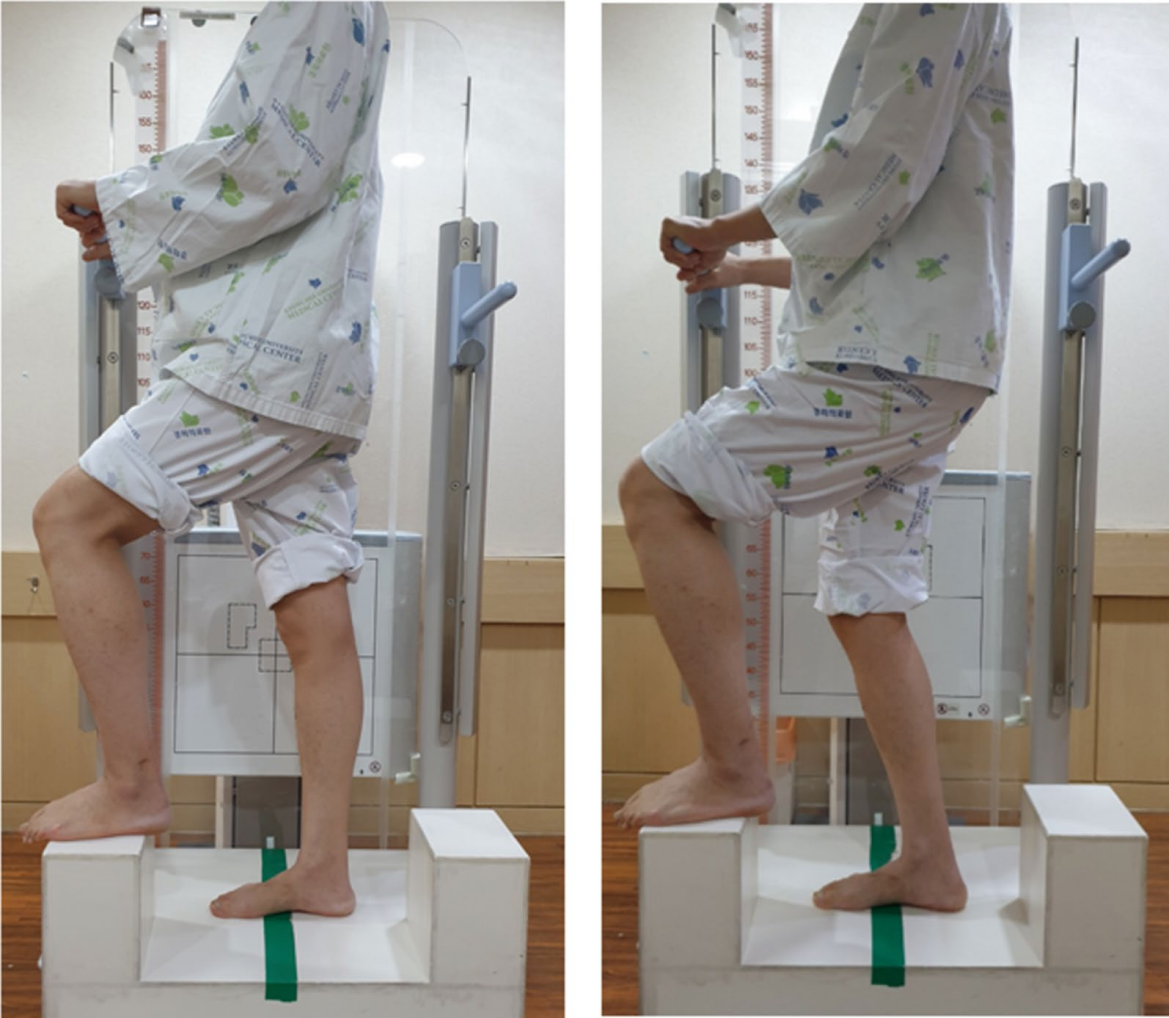
Imaging method of patellar height at knee flexion angles of 0° and 30°

**Fig. 2:**
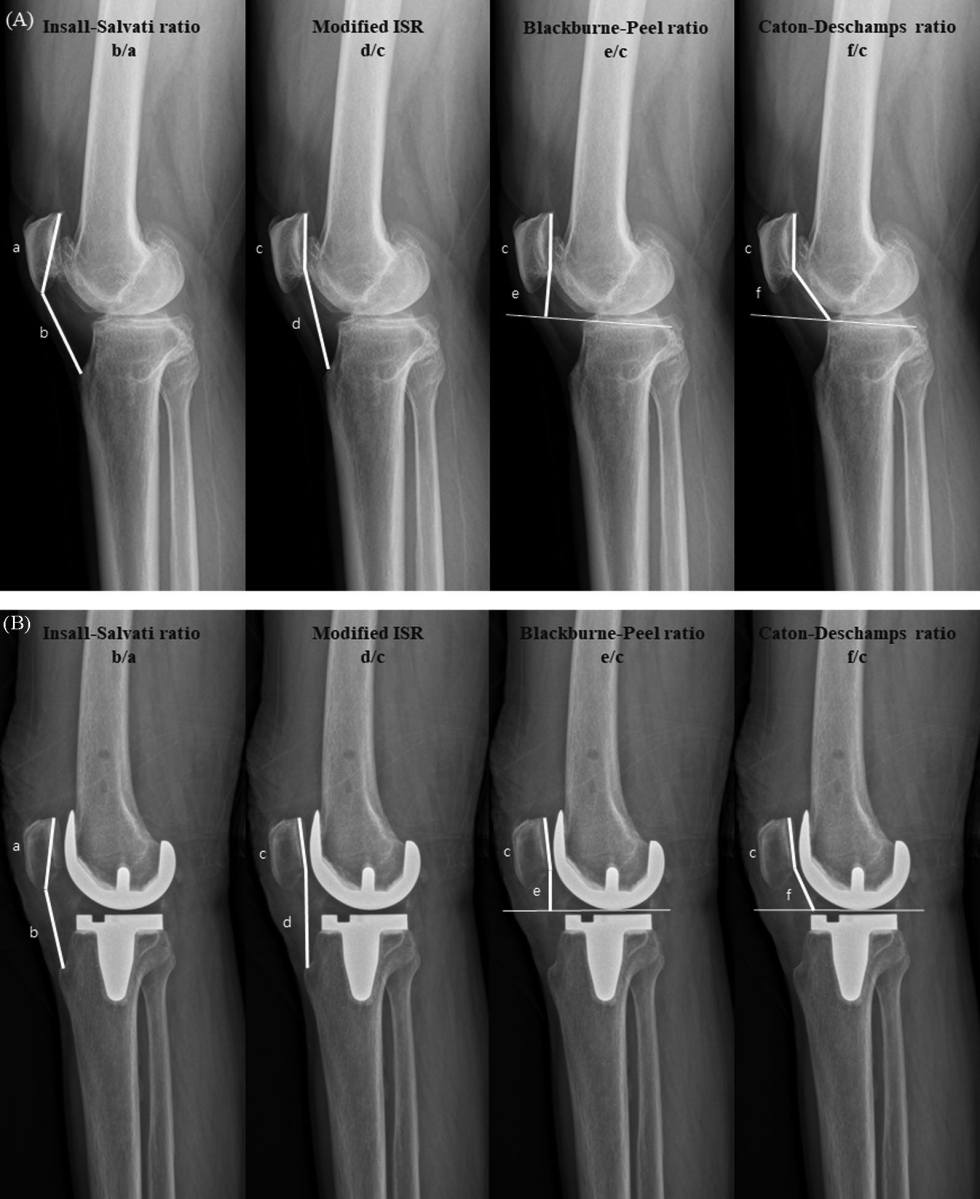
Pre- and postoperative evaluation of patellar height parameters at a knee flexion angle of 0° or 30°. **A** Preoperative evaluation. **B** Postoperative evaluation. **a** longest (diagonal) length of the patella; **b** length of the patellar tendon; **c**, length of the patellar articular surface; **d** distance from the patellar articular surface to the tibial tuberosity; **e** distance between the patellar articular surface and the perpendicular distance to the tangent along the tibial plateau; and **f** distance between the patellar articular surface and the anterior border of the tibial plateau

For the evaluation of the patellar height, other true lateral radiographs were taken at knee flexion angles of 0° and 30° under weight-bearing conditions preoperatively and 3 months postoperatively (Fig. 1). The quality of the true lateral radiograph was confirmed by ensuring the superimposition of the medial and lateral femoral condyles of the distal femur [7]. The ISR, mISR, BPR, and CDR were radiographically measured to properly evaluate the patellar height [5] (Fig. 2).

The images were transferred digitally to a picture archiving and communication system (PACS; Infinitt, Seoul, Korea) and then manipulated for radiographic measurement. Assessments were performed on a 61-cm (24-inch) monitor (SyncMaster 249HM; Samsung, Seoul, Korea) in portrait mode using PACS software. The minimum difference that the software could detect was 0.01 mm. Two independent investigators evaluated all of the radiographic parameters for the patellar height to reduce observation bias. The interobserver reliability of the measurements was assessed using an intraclass correlation coefficient (ICC). The ICC for all measurements was > 0.8, and the average values were used for the study.

### Statistical analysis

The patellar height measured with the ISR, mISR, BPR, and CDR at knee flexion angles of 0° and 30° were compared using the paired *t*-test. The correlations between the patellar height measurements at knee flexion angles of 0° and 30° were analyzed using Pearson correlation analysis. The strength of the correlation was determined by the absolute values of the correlation coefficient: 0–0.19 was regarded as very weak, 0.2–0.39 was regarded as weak, 0.40–0.59 was regarded as moderate, 0.6–0.79 was regarded as strong, and 0.8–1 was regarded as a very strong correlation [8]. The degree of agreement between the measurements at knee flexion angles of 0° and 30° was analyzed using the limit of agreement (LoA) of the Bland–Altman analysis; a difference of <  ± 0.2 between the measurements at knee flexion of 0° and 30° was deemed clinically acceptable [9]. Statistical analyses were performed using SPSS software (ver. 20.0; SPSS, Inc., Chicago, IL, USA); *p*-values < 0.05 were considered to indicate statistical significance.

A power analysis was performed to determine the minimum sample size affording sufficient power, with the variables of which the LoA was identified to be lower than clinically acceptable criteria (± 0.2) in the Bland Altman analysis. The power and alpha level were set to 0.8 and 0.05, respectively. As a result, the maximum appropriate sample size necessary for the significant variables was 98 cases. Therefore, it was determined that our sample size was adequately powered.

## Results

**Fig. 3:**
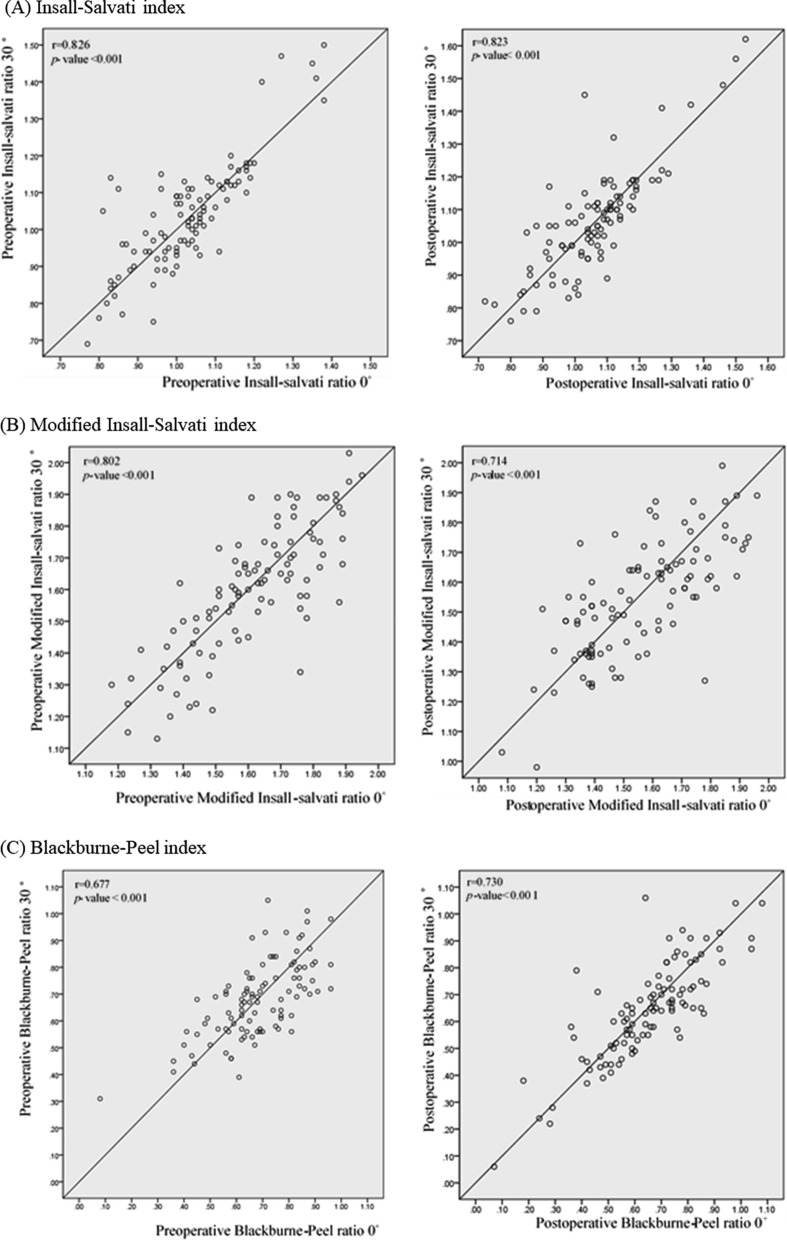

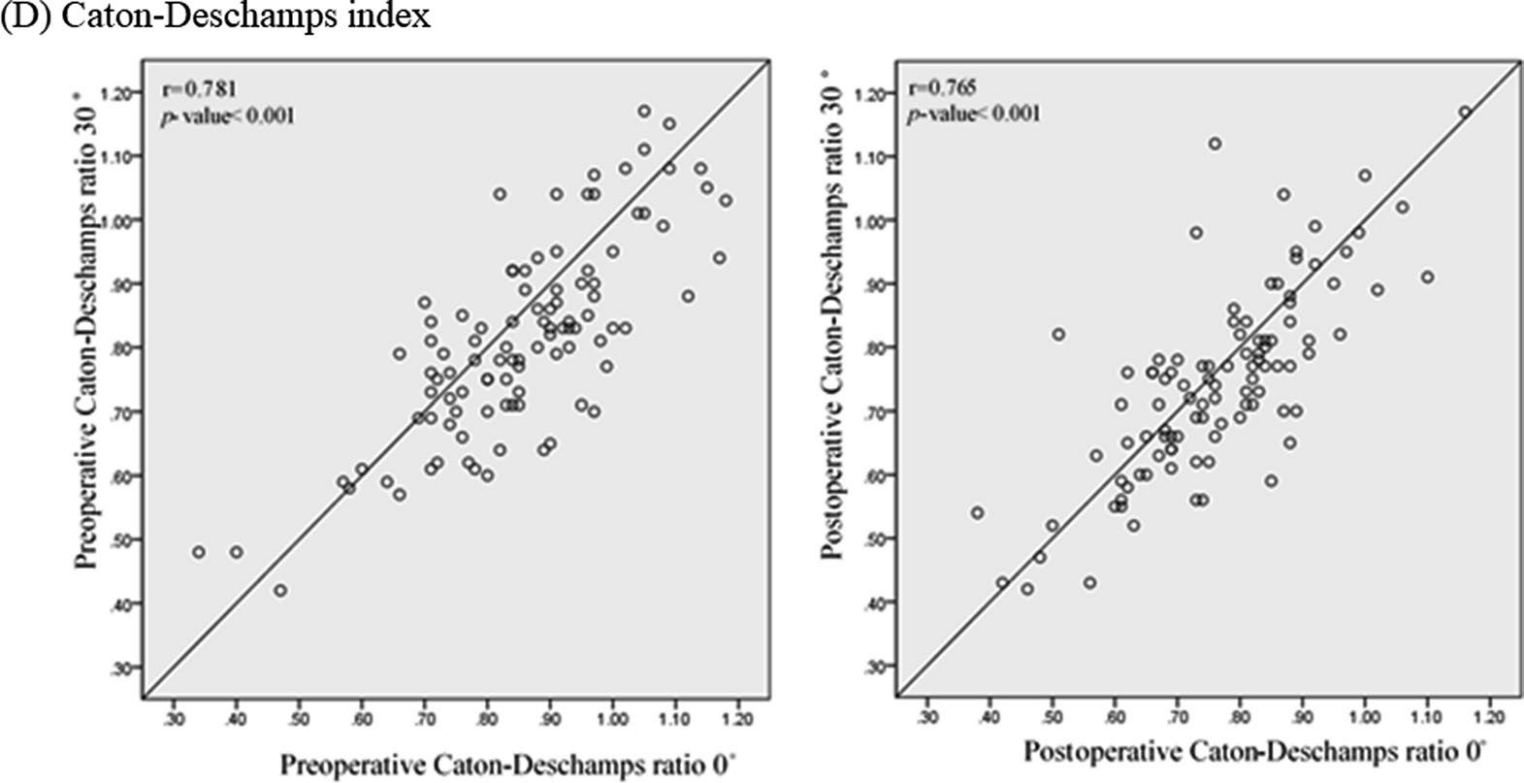
Correlation of pre- and postoperative parameters of patellar height between knee flexion angles of 0° and 30°. **A** Insall–Salvati ratio. **B** Modified Insall–Salvati ratio. **C** Blackburne–Peel ratio. **D** Caton–Deschamps ratio

No significant differences were observed between the patellar height measured at knee flexion 0° and 30° (Table 2). There were very strong correlations between the pre- and postoperative ISR (*r* = 0.826 and 0.823, respectively, *p* < 0.001), and the preoperative mISR (*r* = 0.802, *p* < 0.001) between the knee flexion angles of 0° and 30° (Fig. 3). Strong correlation was observed in the other pre- and postoperative measurements (*r* = 0.677–781) (Fig. 3).

**Table 2:** Radiographic results, including patella height

	0°	30°	*p*-value
Preoperative			
Insall–Salvati index	1.03 ± 0.13	1.04 ± 0.15	0.268
Modified Insall–Salvati index	1.60 ± 0.18	1.60 ± 0.20	0.328
Blackburne–Peel index	0.68 ± 0.15	0.68 ± 0.14	0.850
Caton–Deschamps index	0.85 ± 0.15	0.84 ± 0.15	0.233
Postoperative			
Insall–Salvati index	1.06 ± 0.14	1.06 ± 0.16	0.686
Modified Insall–Salvati index	1.56 ± 0.20	1.54 ± 0.19	0.294
Blackburne–Peel index	0.65 ± 0.18	0.64 ± 0.18	0.429
Caton–Deschamps index	0.76 ± 0.14	0.75 ± 0.15	0.328

**Fig. 4:**
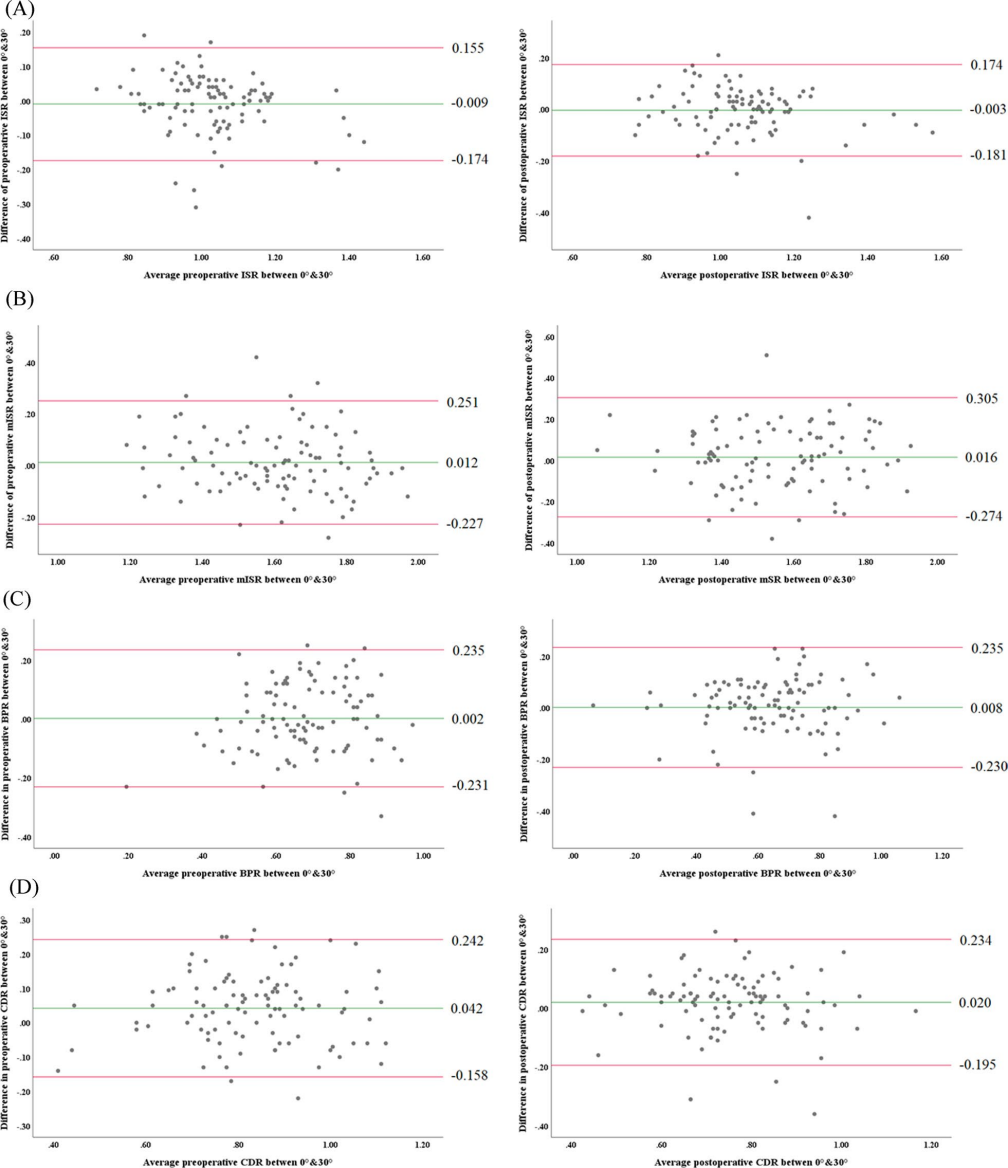
Bland–Altman plot of pre- and postoperative parameters of patellar height between knee flexion angles of 0° and 30°. **A** Pre- and postoperative Insall–Salvati ratio. **B** Pre- and postoperative modified Insall–Salvati ratio. **C** Pre- and postoperative Blackburne-Peel ratio. **D** Pre- and postoperative Caton-Deschamps ratio. The green line indicates the average difference in the measurements between knee flexion at 0° and 30°. The upper and lower red lines indicate the 95% confidence interval range of the difference in the measurements between knee flexion at 0° and 30°

Regarding the agreement, the pre- and postoperative ISR was considered interchangeable between 0° and 30°, while the other indices were not. All of the average for difference in the patellar height measurements between knee flexion angles of 0° and 30° were < 0.05 (−0.009 to 0.042) (Fig. 4). The range between upper and lower LoAs of the ISR measured at knee flexion of 0° and 30° was <  ± 0.20 preoperatively (−0.174 ~ 0.155) and postoperatively (−0.181 ~ 0.174). The range between upper and lower LoAs for all other pre- and postoperative measurements did not lie within the clinically acceptable range (Fig. 4).

## Discussion

The most important finding of this study was that the correlation between the ISR measured at 0° and 30° of knee flexion under weight-bearing was very strong, both before and after TKA. Moreover, only the agreement of the ISR measured between 0° and 30° of flexion was clinically acceptable among the four widely used patellar height ratios before and after TKA.

The measurement methods for patellar height can be divided into a direct method refereeing the femur and an indirect method refereeing the tibia [10]. Although direct methods reveal the true relationship of the patella to the femur, their accuracy depends on the knee flexion angle owing to femoral rollback and rotation [7]. Indirect methods are less susceptible to the knee flexion angle, and are the most widely accepted for clinical use [7]. The four measurement methods in this study belong to the indirect method, and we expected that the correlation and agreement between measurements at knee flexion angles of 0° and 30° would be adequate. Narkbunnam and Chareancholvanich [7] also reported that varying the degree of knee flexion (0°, 30°, and 60°) under nonweight bearing did not result in clinically important effects across the four measurement methods, despite small statistical differences. Additionally, the patella tendon can be sufficiently stretched owing to the action of the quadriceps muscle in weight bearing knee extension [7, 11]. A previous study reported that the contraction of the quadriceps upon weightbearing resulted in statistically significant proximal displacement of the patella when the ISR, mISR, BPR, and CDR were measured [11]. Reebonlap et al. [12] reported that there was no clinically significant difference in patellar tendon length evaluated on weight-bearing lateral radiographs at 0° and 30° flexion. Accordingly, our findings demonstrated no significant differences in patellar height measurements between 0° and 30° of knee flexion under weight-bearing. Moreover, the correlation between the values measured at the two angles of knee flexion was at least strong in all of the methods.

**Fig. 5:**
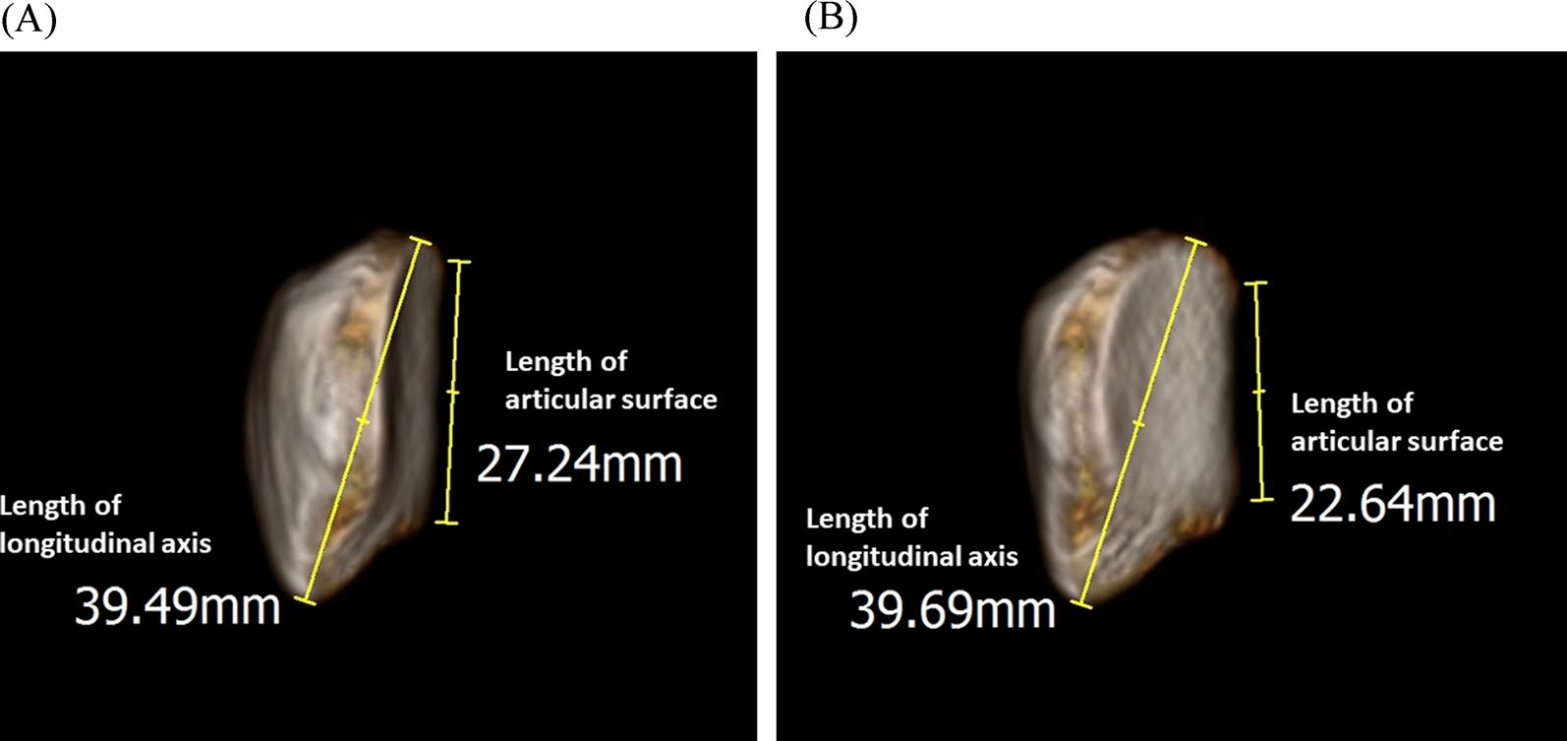
Length of the patellar articular surface and the longitudinal axis shown in the 2-dimensional sagittal angle. **A** Patella with no tilt.at 0° of knee flexion. **B** Patella with a medial tilt 5°at 30° of knee flexion. When the patellar tilt changes at 30° of knee flexion, the length of the articular surface shown in the 2-dimensional sagittal plane of the lateral radiograph can appear different from that at 0° flexion. However, the length of the longitudinal axis connecting the superior and inferior poles of the patella remains relatively constant in the sagittal plane, regardless of tilt

However, the agreement was clinically acceptable only in the ISR. The common difference between the ISR and other measurement methods is that the ISR refers to the longitudinal axis of the patella and the others refer to the patellar articular surface. It is known that the axial alignment of the patella changes in the direction of medial tilting during early flexion up to 30° [13]. A supero-inferior length of the patellar articular surface depends on the sagittal plane; therefore, when the patellar tilt changes at 30° of knee flexion, the length of the articular surface shown in the 2D sagittal plane of the lateral radiograph can appear different from that at 0° flexion (Fig. 5). This is thought to explain why the agreement between the measurements at 0° and 30° was not clinically acceptable in the mISR, BPR, and CDR. However, even if the patella tilt changes, the length of the longitudinal axis connecting the super and inferior poles of the patella does not seem to change significantly even in the 2D sagittal plane (Fig. 5). This mechanism may be reason why the correlation between ISR measured at 0° and 30° flexion was very strong, and degree of agreement between the measurements in the two flexion angles was acceptable in the ISR.

The criterion for the clinical acceptance of the LoA is subjectively determined by a researcher using Bland–Altman analysis [14]. The criterion for ISR in the present study was set as ± 0.2 with reference to the previous study [9], which we believe to be clinically reasonable. Nonetheless, it should be acknowledged that this criterion remains somewhat arbitrary and has not been universally validated. From a clinical standpoint, a variation of 0.20 is unlikely to affect clinical decision-making in most patients, given that the range between the ISI thresholds for severe pathologic patellar height is wider; an ISI greater than 1.3–1.5 is considered severe patella alta, while an ISI less than 0.75 is regarded as severe patella baja [15–19]. However, in cases where the measurement lies close to the pathological cut-off, such a difference may influence the interpretation of patellar height and could potentially affect a clinical decision. Consequently, the threshold of 0.20 should be regarded as a pragmatic criterion that facilitates analyzing interchangeability between measurements, rather than as an absolute diagnostic boundary.

Several limitations of this study should be noted. First, our study was conducted in patients who underwent TKA for degenerative osteoarthritis with varus deformity. Although a study with such a cohort has the advantage of a greater similarity to clinical practice, where many patients have osteoarthritic pain or are awaiting TKA, there was a possibility of measurement error due to osteophytes and deformed bony anatomy in osteoarthritic knees. Moreover, after TKA, joint line determination is difficult due to PE, which is not visible evidently on the radiograph, and thus, measurement using the joint line might be inaccurate. However, two independent investigators evaluated all of the radiographic parameters to reduce the bias, and the ICC for all measurements was > 0.8. Second, the applicability of the present findings to patients with postoperative patella baja is restricted, as the incidence of true patella baja following primary TKA is rare. In addition, the applicability of our findings to patients with flexion contracture is limited, because these patients were excluded from the study despite flexion contracture being frequently encountered in advanced knee osteoarthritis requiring TKA. To derive more robust and generalizable conclusions, future investigations should specifically include patients with patella baja or flexion contracture. Third, although the power was sufficient, our sample size was relatively small. A larger sample size would improve the validity of our finding. Fourth, the power of the quadriceps muscle affecting the patellar height may not be controlled consistently during weightbearing. However, the degree of quadriceps power will not affect the patellar height significantly when the strength is greater than adequate level for stretching the patellar tendon [7]. Finally, other, more recent methods used to measure patellar height, including the plateau-patellar angle, were not evaluated [20]. However, our four measurements are the most commonly selected and used methods by physicians to date.

## Conclusions

The ISR measured at 0° or 30° of knee flexion during weightbearing can be interchanged reasonably before and after TKA. Although knee flexion of 30° is the standard, lateral radiograph of 0° flexion can be a reasonable alternative for evaluating patellar height using the ISR.

## Data Availability

Not applicable.
